# mTORC1 signaling and primary cilia are required for brain ventricle morphogenesis

**DOI:** 10.1242/dev.138271

**Published:** 2017-01-15

**Authors:** Philippe Foerster, Marie Daclin, Shihavuddin Asm, Marion Faucourt, Alessandra Boletta, Auguste Genovesio, Nathalie Spassky

**Affiliations:** 1 Ecole Normale Supérieure, Institut de Biologie de l'ENS (IBENS), INSERM U1024, and CNRS UMR 8197, PSL Research University, 46 rue d'Ulm, Paris 75005, France; 2 Division of Genetics and Cell Biology, San Raffaele Scientific Institute, Milan 20132, Italy

**Keywords:** Cilia, Ventricular system, mTORC1 pathway, Hydrocephalus

## Abstract

Radial glial cells (RCGs) are self-renewing progenitor cells that give rise to neurons and glia during embryonic development. Throughout neurogenesis, these cells contact the cerebral ventricles and bear a primary cilium. Although the role of the primary cilium in embryonic patterning has been studied, its role in brain ventricular morphogenesis is poorly characterized. Using conditional mutants, we show that the primary cilia of radial glia determine the size of the surface of their ventricular apical domain through regulation of the mTORC1 pathway. In cilium-less mutants, the orientation of the mitotic spindle in radial glia is also significantly perturbed and associated with an increased number of basal progenitors. The enlarged apical domain of RGCs leads to dilatation of the brain ventricles during late embryonic stages (ventriculomegaly), which initiates hydrocephalus during postnatal stages. These phenotypes can all be significantly rescued by treatment with the mTORC1 inhibitor rapamycin. These results suggest that primary cilia regulate ventricle morphogenesis by acting as a brake on the mTORC1 pathway. This opens new avenues for the diagnosis and treatment of hydrocephalus.

## Abstract (precis)

**Highlighted article:** Primary cilia regulate ventricle morphogenesis in mice by modulating the mTORC1 pathway, highlighting a new role for mTOR signalling during brain development.

## INTRODUCTION

The vertebrate brain forms around a ventricular cavity filled with cerebrospinal fluid (CSF) that undergoes dynamic morphological changes during development. The neural tube wall constitutes a pseudostratified epithelium composed of elongated progenitor cells called radial glial cells (RGCs) that progressively generate, directly or indirectly, all neurons and glial cells. The brain ventricular system plays crucial roles in brain development, function and homeostasis, and leads to severe neurological disorders when defective (Lehtinen and Walsh, 2011; Lowery and Sive, 2009). Deciphering the mechanisms that regulate brain ventricle morphogenesis is thus essential to understand neocortical histogenesis and identify new therapeutic targets for these common pathologies.

The cerebral cortex originates from the dorsal telencephalon and is composed of six layers, each containing different classes of neurons. Cortical neurons and glia are formed through asymmetric divisions of RGCs, which are bipolar cells that traverse the width of the epithelium, with a basal domain at the pial surface and a ciliated apical contact at the ventricular surface. The primary cilium is a microtubule-based membrane protrusion, the axoneme of which is nucleated by the basal body, a modified mother centriole. Its dynamics are intimately linked to the progression of the cell cycle; it grows during the G1 phase and is resorbed just before mitosis (Nigg and Stearns, 2011). The primary cilium is an important signaling organelle at all developmental stages and has been shown to be crucial for morphogenetic processes such as brain patterning and tissue homeostasis (Eggenschwiler and Anderson, 2007; Willaredt et al., 2008). The primary cilium is chemosensitive and detects a variety of signaling molecules that are important for development (e.g. Shh, Wnt, Pdgf) (Guemez-Gamboa et al., 2014). In the kidney, it also acts as a mechanosensor that detects shear stress, leading to the downregulation of the mTOR pathway necessary for the proper control of cell size (Boehlke et al., 2010; Orhon et al., 2016). The cerebrospinal fluid secreted by the choroid plexus provides diffusible signals that are essential for the early development of RGC (Lehtinen et al., 2011; Chau et al., 2015; Higginbotham et al., 2013). The apical localization of the primary cilium is thus optimal for detecting chemo- or mechano-sensory signals from the CSF, and thus participates in brain morphogenesis by regulating RGC physiology (Lehtinen and Walsh, 2011).

Ciliopathies are a group of genetic diseases characterized by impaired ciliary function. Common brain pathologies encountered in ciliopathy patients are neurocognitive impairments, epilepsy and hydrocephalus. Hydrocephalus is a complex multifactorial brain disorder leading to reduced cortical thickness and enlarged ventricular cavities due to the accumulation of CSF in the ventricles at postnatal stages. It is one of the most common birth defects (Zhang et al., 2006) and has been correlated with a wide range of neurodevelopmental disorders, including schizophrenia (Shenton et al., 2001). Abnormal brain ventricle enlargement often develops at early postnatal stages as a result of defects in ependymal cilia (Ibanez-Tallon et al., 2003) or the choroid plexus (Banizs et al., 2005; Del Bigio, 2010). It was also shown that postnatal hydrocephalus could result from increased apoptosis and impaired proliferation of cilium-less NG2^+^ and PDGFR-α^+^ neural progenitors (Carter et al., 2012). In this study, ventriculomegaly and hydrocephalus are defined as brain ventricular enlargement at embryonic or postnatal stages, respectively. To date, the mechanisms leading to prenatal ventriculomegaly have remained obscure.

Using conditional mouse mutants, we show that depletion of the primary cilia of RGCs from E10.5 onwards results in progressive enlargement of the lateral ventricles (ventriculomegaly) without affecting brain patterning. This phenotype is associated with progressive enlargement of the surface area of RGC apical endfeet induced by abnormal upregulation of the mTORC1 pathway. In addition, we show that cilia depletion leads to misorientation of the RGC mitotic spindle, which is associated with an increased number of basal progenitors in the somatosensory cortex. The size of the apical endfeet, the increased number of basal progenitors and the ventricular enlargement are all rescued by rapamycin treatment. These results suggest that the mTORC1 pathway controlled by the primary cilium regulates ventricle morphogenesis and corticogenesis, and thus constitutes a new potential therapeutic target for the treatment of hydrocephalus.

## RESULTS

### Ventricular morphogenesis defects in ciliary mutants

**Fig. 1.**
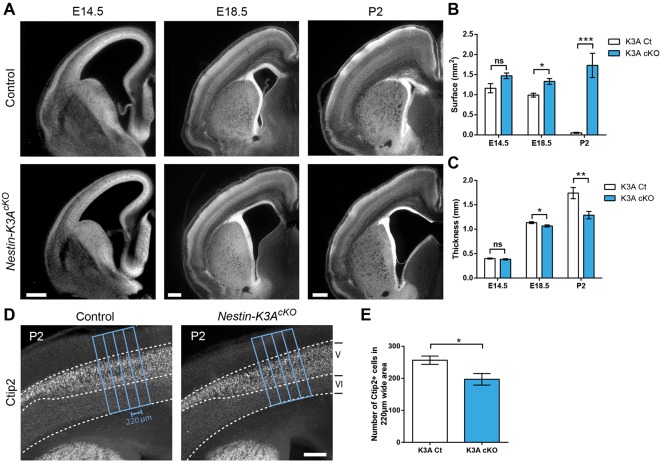
**Ventricular enlargement in ciliary mutants.** (A) Representative coronal sections of control and *Nestin-K3A^cKO^* mutant forebrains at E14.5, E18.5 and P2. (B,C) Concomitant increases in the area of the lateral ventricle and decreases in cortical thickness at each embryonic and postnatal stage show the progression of embryonic ventriculomegaly and postnatal hydrocephalus in the ciliary mutant (*n*=4). (D) Representative Ctip2 immunostaining on coronal sections of control and ciliary mutant forebrains at P2. The boundaries between cortical layers V and VI are indicated by dashed lines. Blue boxes show the area quantified in E. (E) Quantification of the number of Ctip2^+^ cells in the 220-µm-wide area outlined in D (*n*=6). Scale bars: 0.5 mm (A,D). ns, not significant.

During mouse brain development, neuroepithelial (NEP) cells and RGCs extend a primary cilium into the ventricles (Higginbotham et al., 2013; Fig. S1A). The dynamics are tightly correlated with the cell cycle and interkinetic nuclear migration (Kim et al., 2011; Paridaen et al., 2013). To study the role of the primary cilium in forebrain development, we generated conditional ciliary knockouts of floxed *Ift88* or *Kif3a* genes (Haycraft et al., 2007; Marszalek et al., 1999) to ablate cilia from the apical surfaces of either NEP cells (using FoxG1-Cre mice: *FoxG1-K3A^cKO^*) or RGCs (using Nestin-Cre mice: *Nestin-K3A^cKO^* or *Nestin-Ift88^cKO^*, Fig. S1B-E). In *FoxG1-K3A^cKO^* mice, cilia depletion in NEP cells leads to patterning defects and altered GLI3 processing, as previously reported (Benadiba et al., 2012; Besse et al., 2011; Higginbotham et al., 2013; Willaredt et al., 2008; Laclef et al., 2015; Fig. S1F-H). In contrast, no polarity or patterning defects were observed in Nestin-Cre conditional ciliary mutants (*Nestin-K3A^cKO^* or *Nestin-Ift88^cKO^*), in which Cre was expressed in the forebrain from embryonic day (E)11 onwards and recombination leads to complete cilia ablation from E14.5 (Fig. S1B-E). Indeed, both the expression patterns of Ngn2 (Neurog2; a marker of the dorsal telencephalon) and Dbx1 (a marker of the pallium-subpallium boundary), and Gli3 processing were unaltered in Nestin conditional knockout (cKO) mutant forebrains at E12.5 (Fig. S1F-H). Furthermore, the apical positions of the centrosomes (Fig. S1C and Fig. S3A,B) and thus the polarity of RGCs were normal. As previously described (Tong et al., 2014), we observed that *Nestin-K3A^cKO^* conditional ciliary mutants show severe hydrocephalus at postnatal stages (Fig. 1). However, we noticed that at earlier stages (from E18.5 onwards), *Nestin-K3A^cKO^* mice displayed a progressive enlargement of the lateral ventricles (Fig. 1A,B), and reduced cortical thickness (Fig. 1A,C) associated with a moderate but significant decrease in the number of post-mitotic neurons labeled with a Ctip2 antibody (a marker of early-born neurons) (Fig. 1D,E). Similar phenotypes were observed in *Nestin-Ift88^cKO^* mice (data not shown). The enlarged ventricles and the reduced brain tissue observed in the ciliary mutants before motile cilia develop both suggest that ablation of primary cilia at E11 leads to the development of prenatal ventriculomegaly, which might initiate postnatal hydrocephalus.

### Primary cilia abrogation in RGCs leads to apical domain enlargement

**Fig. 2.**
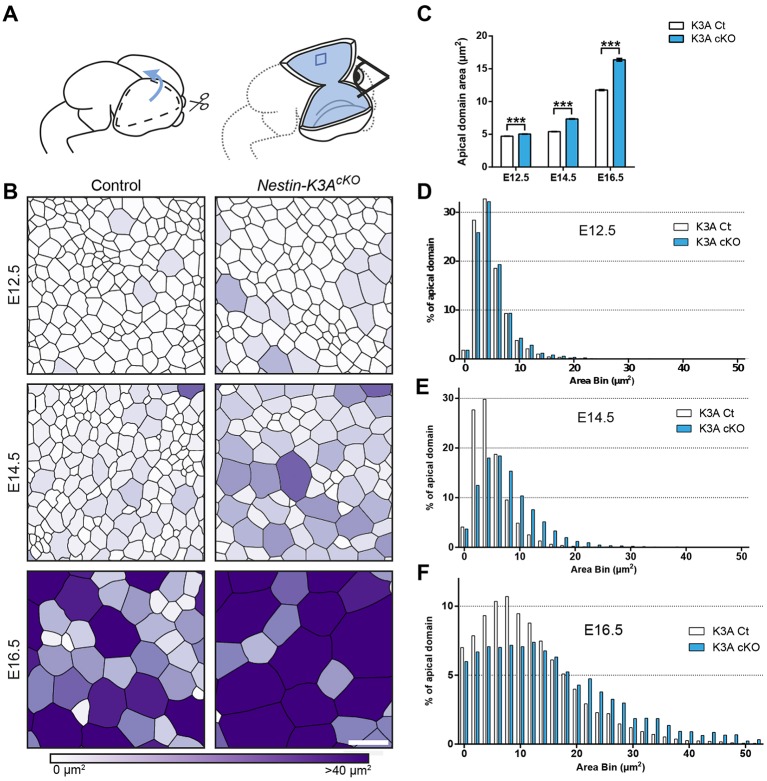
**Cilia abrogation leads to the progressive enlargement of RGC apical domains.** (A) Schematic representation of embryonic forebrain dissection for whole mount preparations of cortical ventricular surfaces. (B) Cortical surfaces immunostained with the ZO-1 antibody shown in Fig. S2A were skeletonized to obtain segmented images of representative cortical surfaces at E12.5, E14.5 and E16.5 of controls and Kif3A ciliary mutants. The surface areas per cell are color coded from white (less than 10 µm^2^) to dark purple (more than 40 µm^2^). (C) Quantification of the surface area of the apical domains in controls (white) and ciliary mutants (blue) at E12.5, E14.5 and E16.5. (D,E,F) Sample distribution of apical domain areas in control (white) and Kif3a^cKO^ (blue) embryos at E12.5 (D), E14.5 (E) and E16.5 (F) (2 µm^2^ bins). Numbers of apical domains measured in B at E12.5 (11805 for controls and 13329 for K3A^cKO^, *n*=3), at E14.5 (12731 for controls and 9173 for K3A^cKO^, *n*=3) and E16.5 (8895 for controls and 7476 for K3A^cKO^, *n*=3). Apical domains measured in C: 100 per genotype blind to the conditions. Scale bar: 5 µm.

Primary cilia extend from the apical surface of RGCs in the brain ventricles. To further identify the cellular mechanisms leading to ventricular enlargement, we analyzed the apical domains of RGCs on whole-mount somatosensory cortical ventricular walls in *Nestin-K3A^cKO^* ciliary mutant and control embryos (Fig. 2A) stained with an antibody against ZO-1 (Tjp1) that labels the tight junctions and delimits individual RGC apical domains (Fig. S2A,B,D). The size of the apical domains was quantified and color-coded with Packing Analyzer (Aigouy et al., 2010) and CellProfiler (Lamprecht et al., 2007) software (Fig. 2B-F). In control embryos, the mean size of the apical domains increased from E12.5 to E16.5; they were more heterogeneous at E16.5 than they were at E14.5 (Fig. 2C-F). Thus, the size of the RGC apical domain increases progressively during normal development, as previously shown (Nishizawa et al., 2007). Interestingly, the increase was significantly greater in ciliary mutants than in controls at all stages analyzed, and the difference increased with age: the relative difference in size between ciliary mutant and controls was 7% at E12.5, 35% at E14.5 and 39% at E16.5 (Fig. 2C-F). Similar results were obtained in *Nestin-Ift88^cKO^* embryos compared with controls (Fig. S2B-C). Finally, as *Nestin* is also expressed in the lateral ganglionic eminence (LGE), we confirmed that apical domain enlargement was also present in *Nestin-K3A^cKO^* lateral ganglionic eminence at E14.5 (Fig. S2D,E). These results suggest that cilia abrogation leads to a progressive increase in the size of RGC apical domains during forebrain development.

### Apical domain enlargement during mitosis is greater in ciliary mutants than in controls

**Fig. 3.**
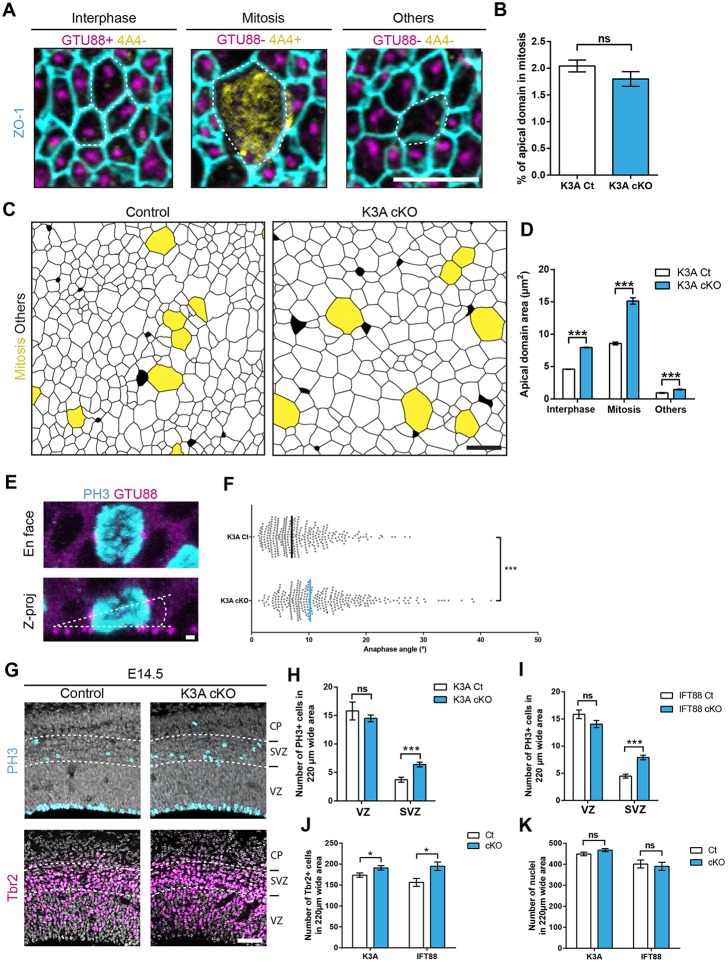
**Apical domain enlargement leads to corticogenesis defects.** (A) Triple immunostaining with ZO1 (cell junctions in cyan), GTU88 (pericentriolar marker in magenta) and 4A4 (mitotic cells in yellow) antibodies on E14.5 whole mount cortical surfaces allowed us to classify the cells in three categories: cells in interphase (GTU88^+^/4A4), cells in mitosis (GTU88^−^/4A4^+^) and other cells (GTU88^−^/4A4^−^). (B) Quantification in controls and *Nestin-K3A^cKO^* mutants of the percentage of 4A4^+^ (mitotic) ventricular cells: the total number of mitotic cells contacting the ventricle (number of apical domains in mitosis) was related to the number of GTU88^−^/4A4^+^ cells. (C) Segmented images of the immunostaining shown in Fig. S3A,B, in which mitotic 4A4^+^ cells are shown in yellow. (D) Quantification of the apical domain areas of control (white) and mutant mice (blue) in each cell category. Data are the mean±s.e.m. *n*=3 per genotype per experimental condition in B and D. Numbers of apical domains analyzed in D in interphase (20279 for controls and 12843 for *Nestin-K3A^cKO^*), mitotic (432 for controls and 245 for *Nestin-K3A^cKO^*) and others (604 for controls and 668 for *Nestin-K3A^cKO^*) cells. (E) *En face* view and *Z*-projection of a cilium-less apically positioned PH3^+^ mutant cell double-immunostained with the GTU88 antibody illustrating the methodology for measuring anaphase spindle orientation. The ventricular surface is delineated by the positions of GTU88^+^ dots in neighboring PH3^−^ cells (angle between dashed lines). (F) Quantification of the angle formed by a line passing through both centrosomes of cilium-less and control PH3^+^ cells and the ventricular surface. Anaphase angles measured in L: 324 per genotype. (G) Representative images of PH3^+^ mitotic cells and Tbr2^+^ intermediate progenitors in coronal sections of the somatosensory cortex of *Nestin-K3A^cKO^* mutant mice and controls at E14.5. (H,I) Quantification of PH3^+^ mitotic cells in the SVZ on a 220-µm-wide area from *Nestin-K3A^cKO^* and *Nestin-Ift88^cKO^* mice shows a significant increase in the mutants compared to the controls. (J,K) Quantification of Tbr2^+^ intermediate progenitor cells and DAPI^+^ cells in 220-µm-wide areas of *Nestin-K3A^cKO^* and *Nestin-Ift88^cKO^* mice and their respective controls. Data are the mean±s.e.m. in B,D,H-K or median in F (*n*=3 per genotype). Scale bars: 5 µm (A,C); 1 µm (E); 50 µm (G).

RGCs are highly polarized cells in which nuclear interkinetic migration takes place in the ventricular zone (VZ). Their nucleus is larger than the surface of their apical domain, suggesting that the size of the apical domain reflects the distance of the cell body from the ventricle and, thus, the phase of the cell cycle. To examine this possibility and determine whether the increase in the size of the apical domain in cilia mutants is correlated with a given phase of the cell cycle, whole-mount cortical ventricular walls from ciliary mutants and controls were triple-labeled with antibodies against ZO-1, 4A4 (phospho-vimentin; cytoplasmic mitotic marker) and γ-tubulin (Tubg1; marker of the pericentriolar material). Apical domains in three categories of cells were examined: interphase cells (γ-tubulin^+^/4A4^−^), mitotic cells (γ-tubulin^−^/4A4^+^) and other cells (γ-tubulin^−^/4A4^−^) (Fig. S3A,B, Fig. 3A-D). The size of the apical domains is significantly smaller during the interphase than during mitosis both in controls and *Nestin-K3A^cKO^* mutants, suggesting that its size is precisely controlled as the cell cycle advances. The percentage of mitotic cells was similar in controls and mutants (Fig. 3A,B), in accordance with the observation that the number of mitotic cells in the ventricular zone was unaffected in cilium-less RGCs (see below, Fig. 3G-I). However, the size of the apical domains in both interphase and mitotic cells was greater in mutants than in controls, suggesting that cilia abrogation leads to a similar enlargement of both interphase and mitotic apical domains (Fig. S3A,B, Fig. 3C,D).

### Apical endfoot enlargement in ciliary mutants is associated with spindle orientation defects

Cell shape and mechanical cues have been shown to guide division orientation in epithelia (Wyatt et al., 2015) and mitotic spindle orientation contributes to cell fate determination (Paridaen and Huttner, 2014). We therefore tested whether RGC apical domain enlargement in ciliary mutants could perturb mitotic spindle orientation. We measured anaphase spindle orientation in controls and *Nestin-K3A^cKO^* mutants. Subtle defects in spindle orientation were observed: the median at E12.5 was 6.2° in controls but 8.7° in mutants (Fig. S3C). This defect worsened at E14.5: the median was 6.9° in controls and 10.2° in mutants (Fig. 3E,F). To test whether these defects affected other cells in the cortex, E14.5 coronal sections were immunostained with antibodies against PH3 (Phc3; mitotic cells), Tbr2 (Eomes; basal progenitors, BPs) and Ctip2 (Bcl11b; early born neurons). Although the abundance of mitotic or Pax6^+^ (RGC) cells was similar in the VZ of controls and *Nestin-K3A^cKO^* and *Nestin-Ift88^cKO^* mutants (Fig. 3G-I, Fig. S3D-F), the number of mitotic cells increased 2-fold in the subventricular zone (SVZ) of the ciliary mutants compared with controls and was associated with a significant increase in the number of basal progenitors (Fig. 3G-K). These results suggest that cilia abrogation leads to apical domain enlargement, impaired mitotic spindle orientation, increased cell proliferation and the generation of supernumerary progenitors in the SVZ at E14.5.

Other non-ciliary effects of Kif3a or Ift88 knockout have been reported to affect microtubule dynamics, leading to spindle misorientation (Delaval et al., 2011; Kodani et al., 2013; Teng et al., 2005); ninein (Nin)^+^ sub-distal appendages were absent from Kif3a embryonic knockout mouse fibroblasts (Kodani et al., 2013) and removal of ninein from the developing neocortex caused premature depletion of progenitors from the VZ (Wang et al., 2009) and enlargement of the endfoot area of apical progenitors (Shinohara et al., 2013). Here, no defects in subdistal appendages or ninein localization at the centrosome were detected in cilium-less RGCs at E14.5 (Fig. S4A,B). Similarly, no defects in polarity were observed in cortical ventricular walls labeled with adherens junction markers on *en face* views of the RGCs lacking a primary cilium, suggesting that cilia abrogation did not affect markers of apico-basal cell polarity (Fig. S4C). Thus, cilia abrogation in these ciliary mutants does not lead to the loss of sub-distal appendages. Mitotic spindle misorientation observed in *Nestin-K3A^cKO^* ciliary mutant is probably due to increased cell size rather than impaired attachment of microtubules to the centrosome. These results, together with the similar phenotype observed in two different ciliary mutants (Kif3a and Ift88), suggest that mitotic spindle misorientation might be directly linked to cilia abrogation.

The number of Ctip2^+^ neurons at E14.5 was similar in controls and ciliary mutants (Fig. S3D-F) but it was decreased at postnatal day (P)2 (Fig. 1D,E). Interestingly, no defects in cell survival could be observed at either age in controls and *Nestin-K3A^cKO^* mutant mice, suggesting that the decrease in Ctip2^+^ cells at P2 was not due to increased cell death. Indeed, the numbers of activated caspase-3^+^ cells (data not shown) and TUNEL^+^ cells were similar in *Nestin-K3A^cKO^* neonatal mice (7±2 TUNEL^+^ cells per section) and controls (6±1 TUNEL^+^ cells per section). These results thus confirm previous findings that cilia abrogation in *Nestin-K3A^cKO^* mice does not lead to increased cell death (Tong et al., 2014). To test whether thinning of the cortex could be due to tissue stretch, we quantified cell density on tissue sections at P2. However, no significant differences were observed (Fig. S4E). Altogether, these results suggest that the thinner cortex at P2 is due to premature differentiation rather than defects in cell survival or changes in cell density.

### Cilia regulate RGC apical domain size through the mTORC1 pathway

**Fig. 4.**
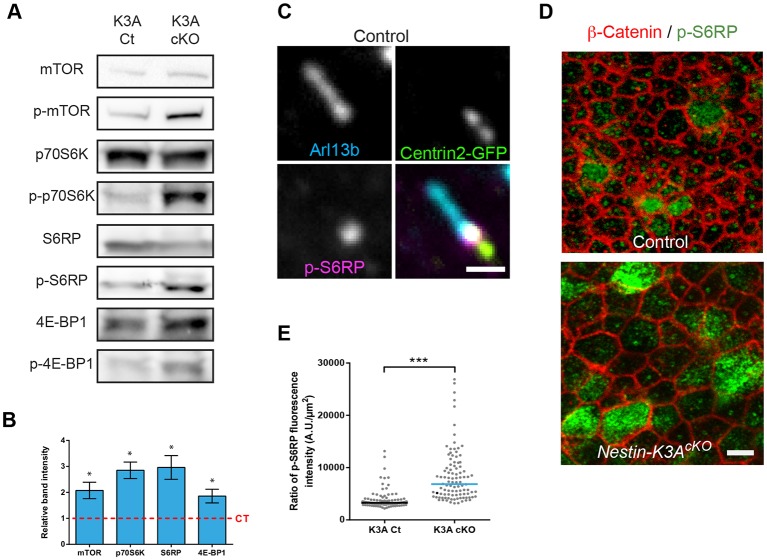
**Cilia abrogation leads to an increase in the mTORC1 pathway.** (A) Western blot analysis of E14.5 control and cilium-less cortical lysates shows increased phosphorylation of mTOR targets in ciliary mutants compared with controls. (B) Quantification of bands from three independent experiments (A) shows that the increase in mTOR pathway activity in ciliary mutants compared with controls is significant. (C) Double immunostaining of an E14.5 Centrin-2-GFP control cortical ventricular surface with Arl13b (cilia, cyan), and phosphorylated S6RP (p-S6RP, magenta) antibodies showing p-S6RP staining at the mother centriole of the centrosome in ciliated cells. (D) Double immunostaining of E14.5 cortical surfaces from control and *Nestin-K3A^cKO^* ciliary mutants with antibodies against β-catenin (red) and p-S6RP (green). (E) Quantification of the ratio between the intensity of p-S6RP fluorescence and the surface area of the apical domain in controls and *Nestin-K3A^cKO^* ciliary mutants at E14.5 shows an increase in the level of p-S6RP in the mutants. Apical domains measured in E: 100 per genotype blind to the condition. Scale bars: 1 µm (C) and 5 µm (D).

In the kidney, deletion of the primary cilium by knockout of Kif3a caused cell enlargement as a result of abnormal upregulation of the mTORC1 pathway (Boehlke et al., 2010; Orhon et al., 2016). To test whether cilia regulate the size of the RGC apical domain through the mTORC1 pathway, we analyzed dorsal telencephalic lysates from controls and ciliary mutants, at E14.5, for phosphorylation of mTOR and its targets p70S6K, S6RP (S6 ribosomal protein) and 4E-BP1, which are established markers of mTORC1 activity. The levels of phosphorylated mTOR and downstream targets increased significantly in ciliary *Nestin-K3A^cKO^* mutants compared with controls (Fig. 4A,B, Fig. S5A). Immunostaining of the downstream mTORC1 effector with an antibody against phosphorylated ribosomal S6 protein (p-S6RP) revealed its localization at the mother centriole of the centrosome and at the apical surface of mitotic RGCs (Fig. 4C,D). Interestingly, ciliary mutant apical domains contained more p-S6RP than controls, as shown by the increased intensity of p-S6RP fluorescence at the mutant ventricular surface (Fig. 4E).

**Fig. 5.**
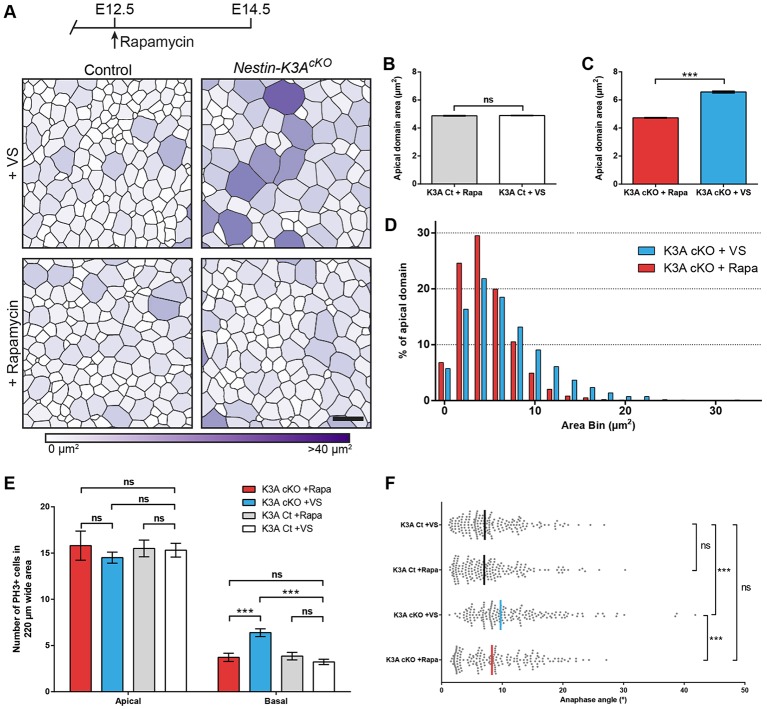
**Apical domain enlargement and corticogenesis defects at E14.5 are rescued by a single rapamycin injection at E12.5.** (A) Cortical surfaces immunostained with ZO-1 antibody shown in Fig. S5B were skeletonized to obtain segmented images of representative cortical surfaces at E14.5 from controls and *Nestin-K3A^cKO^* ciliary mutants injected with rapamycin or vehicle solution. Color code is as described in Fig. 2. (B,C) Quantification of apical domain areas at E14.5 shows a significant decrease in rapamycin-injected (red bar in C) compared with vehicle-injected ciliary mutants (blue bar in C); no significant difference is observed between rapamycin- and vehicle-injected controls (B). (D) Distribution of apical domain surfaces in cortices from E14.5 mutants injected with vehicle (blue) or rapamycin (red). (E) Quantification of the relative number of PH3^+^ cells in 220-µm-wide areas on coronal sections at E14.5 in rapamycin- and vehicle-injected controls and ciliary mutants shows significant rescue of the number of basally positioned PH3^+^ cells, which return to normal levels in rapamycin-injected ciliary mutants but not in controls. (F) Quantification of anaphase angles of PH3^+^ apical cells in control and ciliary mutants injected with rapamycin or vehicle show significant rescue of the mitotic spindle misorientation in rapamycin-injected ciliary mutants. Data are the mean±s.e.m. in B-E or the median in F (*n*=3 per genotype per experimental condition). Apical domain analyzed in B-D for vehicle (14040 for controls, 14001 for *Nestin-K3A^cKO^*) and rapamycin treatment (14900 for controls, 14399 for *Nestin-K3A^cKO^*). Anaphase angles measured in F: 180 per condition. Scale bar: 5 µm.

To further examine whether the mTOR pathway regulates the size of the RGC apical domain, we injected the mTORC1 inhibitor rapamycin (6 mg/kg) or vehicle intraperitoneally at E12.5. In control embryos, rapamycin injection at E12.5 had no significant effect on the size of RGC apical domain (Fig. 5A,B, Fig. S5B). In *Nestin-K3A^cKO^* embryos, in which the size of RGC apical domains increased by 35% compared with controls (Fig. 2C), rapamycin injection at E12.5 led to an apical domain size comparable to that observed in control embryos at E14.5 (Fig. 5A-D). These results suggest that rapamycin treatment prevents excessive apical domain enlargement at E14.5 in *Nestin-K3A^cKO^*, which might result from ineffective downregulation of mTORC1 due to the absence of primary cilia. Interestingly, the misorientation of the mitotic spindle and the number of PH3^+^ basal mitotic figures in the developing somatosensory cortex was also rescued in cilium-less RGCs of *Nestin-K3A^cKO^* embryos at E14.5 after a single injection of rapamycin at E12.5 (Fig. 5E,F). These results thus show that cilia abrogation in developing RGCs leads to an abnormal increase in the size of apical domains as a result of upregulation of the mTORC1 pathway, which leads to spindle misorientation in apical mitotic cells and to an abnormal increase in the number of PH3^+^ basal mitotic figures.

### Successive rapamycin injections rescue the ventriculomegaly phenotype

**Fig. 6.**
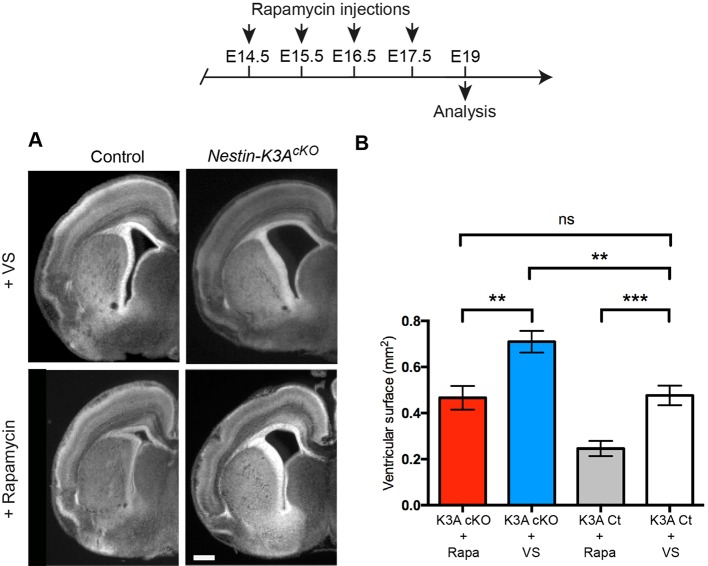
**Ventriculomegaly phenotype at E19 is rescued by repeated rapamycin injections from E14.5 to E17.5.** (A) Representative coronal sections of control and *Nestin-K3A^cKO^* mutant forebrains at E19. (B) Quantification of the area of the lateral ventricle in control and ciliary mutants injected with rapamycin or vehicle solution from E14.5 to E17.5 show the rescue of the ventriculomegaly phenotype after successive injections of rapamycin. Scale bar: 500 μm.

Since the increase in size of the cells can be rescued by rapamycin administration, to further confirm that progressive enlargement of RGC apical domains leads to ventriculomegaly, we injected rapamycin at a low dose (1 mg/kg) once a day between E14.5 and E17.5 and analyzed the size of the lateral ventricles at E19. This low dose of rapamycin was used because we observed a high rate of mortality with consecutive treatments at higher doses. Interestingly, four successive injections of rapamycin rescued the size of the ventricle in ciliary mutants, which was not significantly different from that of control embryos injected with vehicle (Fig. 6). Furthermore, although a single injection of rapamycin had no effect on controls at E14.5 because the mean area of the apical domain was similar at E12.5 and E14.5 (4.7 µm^2^ at E12.5 and 4.8 µm^2^ at E14.5; Fig. 2C and Fig. 5A,B), we observed that consecutive rapamycin injections significantly decreased the size of the ventricles in control embryos (Fig. 6), suggesting that mTORC1 inhibition by rapamycin treatment acts as a brake on the enlargement of RGC apical domains but does not decrease the size of the apical domains. These results suggest that the progressive increase in RGC apical domain size during normal cortical development contributes to ventricle morphogenesis and that it is regulated by the mTOR pathway (Fig. 2), although rapamycin treatment might also affect other cell types surrounding the ventricles (Magri and Galli, 2013).

## DISCUSSION

In the present study, we genetically ablated the primary cilium of RGCs using Ift88 or Kif3a conditional mutant mice and observed a progressive increase in the size of their ventricular apical domains through upregulation of the mTORC1 pathway. These defects are associated with a significant perturbation of their mitotic spindle orientation, an overproduction of basal mitotic progenitors at E14.5, a decreased number of cortical neurons at later stages and progressive enlargement of the lateral ventricles, defined as ventriculomegaly. Interestingly, all these defects were attributable to an abnormal increase in mTORC1 pathway activity as they were rescued by one or several injections of the mTORC1 inhibitor rapamycin. Altogether, this study shows for the first time that the primary cilium of RGCs regulates ventricular morphogenesis through control of the size of their ventricular contact by acting as a brake on the mTORC1 signaling pathway.

Here, we used conditional knockout of ciliary genes (*Ift88* and *Kif3a*) in *Nestin-Cre* mice to deplete RGCs of their primary cilium, bypassing its role in brain patterning and the polarity of neuroepithelial cells (Benadiba et al., 2012; Besse et al., 2011; Higginbotham et al., 2013; Willaredt et al., 2008; Wilson et al., 2011). Although Nestin-Cre expression starts around E10.5 in the forebrain, cilia abrogation in the VZ was complete only at E14.5, as previously shown (Liang et al., 2012; Fig. S1A-E). The phenotypes observed at E12.5 might thus be partially due to inefficient recombination before E12.5. Moreover, Nestin is broadly expressed at later developmental stages, suggesting that some of the phenotypes observed in ciliary mutants are, in part, due to non-cell-autonomous effects.

During their development from RGCs (Spassky et al., 2005), multiciliated ependymal cells enlarge their apical domain (Mirzadeh et al., 2010). It is thus possible that the enrichment of ciliary mutants in large apical domains at a given developmental stage corresponds to premature ependymal differentiation. However, the numbers of ventricular CD24^+^/centrin-2 (Cetn2)-GFP^+^ cells at E18.5 were similar in controls and ciliary mutants, ruling out this hypothesis (Fig. S4D). Another possibility is that depletion of cilia leads to defects in the cytoskeleton and adherens junctions, resulting in increases in ventricular cell delamination and available space at the ventricular surface, and ultimately to enlargement of the apical domains. However, no defects in either adherens junctions or actin cytoskeleton were observed by immunostaining the cortical ventricular surface during corticogenesis in mutant RGC (Fig. S4C). Altogether these observations suggest that ventriculomegaly is due to apical domain enlargement of RGC before their differentiation into ependymal cells, ruling out a contribution of ependymal dysfunction during embryogenesis. The prenatal ventriculomegaly described here precedes and might contribute to the initiation of hydrocephalus observed at postnatal stages (this study and Tong et al., 2014). Postnatal hydrocephalus can thus be caused by a variety of factors including defects in ventricular morphogenesis (this study), disturbed CSF flow due to impaired ependymal cilia function, over-production of CSF by the choroid plexus (Del Bigio, 2010) or combinations of these factors.

In control embryos, RGC apical domains are highly diverse in size at a given developmental stage. The sizes increase throughout development, leading to greater size diversity at later stages (Nishizawa et al., 2007; this study). Enlargement is also observed in ciliary mutants and is greater than in control embryos. Further studies should determine the effects of apical domain morphology, mode of division and long-term potentials on the mechanical constraints exerted by RGCs on the morphogenesis of brain ventricles. We have shown here that the enlargement of the RGC apical domain is due to rapamycin-sensitive control of localized cell growth by the primary cilium. mTORC1 signaling, the targets of which are enriched at the apical domain of RGCs, is known to control cell growth by upregulating protein synthesis (Fig. 4A). This raises the question whether local protein synthesis is involved in apical cell enlargement. Although the size of RGC apical domains has not been studied, microcephaly and ventricular enlargement have been reported in mouse mutants in which the activity of the mTOR pathway was, respectively, defective or aberrantly elevated (Cloetta et al., 2013; Way et al., 2009; Magri et al., 2011). Mechanosensory proteins polycystic kidney disease 1 (Pkd1) and Pkd2 are expressed in primary cilia of RGCs, and their ablation leads to postnatal hydrocephalus and planar cell polarity defects in mouse ventricular epithelium (Wodarczyk et al., 2009; Ohata et al., 2015). Moreover, it was shown that Pkd1 can inhibit the mTOR pathway and regulate cell size in the kidney (Distefano et al., 2009). We thus tested whether Pkd1 contributed to the control of the mTOR pathway and cell size in RGCs by measuring the size of the apical domains in *Nestin-Cre* conditional knockouts of floxed *Pkd1*. However, no difference in the surface area of RGC apical domains was observed at E14.5 (Fig. S6), suggesting that primary cilia do not regulate the size of RGCs through the mechanosensory protein Pkd1 via the mTOR pathway. Interestingly, it was recently discovered that primary cilium-dependent autophagy regulates kidney epithelial cell size through the LKB1-AMPK-mTOR signaling pathway in response to fluid flow (Orhon et al., 2016). Whether the mTOR pathway and the mechanical stress pathway are coordinated in the control of RGC size in the primary cilium-dependent autophagy pathway is an attractive hypothesis that remains to be investigated. Further studies are thus required to identify the upstream signals of the mTOR pathway in these cells. Altogether, our results shed light on a new role of the primary cilium in the local control of cell growth and ventricular morphogenesis during brain development.

## MATERIALS AND METHODS

### Mouse strains

All animal care was in accordance with French and European legislation. All the mice used in this study were previously described: *Kif3a^fl^*, *Kif3a^ko/+^* (Marszalek et al., 1999), *IFT88^fl^*, *IFT88^ko/+^* (Haycraft et al., 2007), *Nestin-Cre* (Tronche et al., 1999), *FoxG1-Cre* (Hébert and McConnell, 2000), *Rosa26^mTmG^* (Muzumdar et al., 2007), *Centrin-2-GFP* (Higginbotham et al., 2004). To produce conditional Kif3a and IFT88 mutants, we crossed *Kif3a^fl/fl^* or *IFT88^fl/fl^* mice with *Kif3a^ko/+^ Nestin-Cre^+/−^* or *IFT88^ko/+^ Nestin-Cre^+/−^* mice, respectively. To obtain conditional *Kif3a FoxG1* mutants, we crossed *Kif3a^fl/fl^* with *Kif3a^ko/+^ FoxG1-Cre^+/−^*. *Nestin::Cre;Kif3a^ko/fl^*, *Nestin::Cre;Ift88^ko/f^*^l^ and *Nestin::Cre;FoxG1^ko/fl^* were used as ciliary mutants, whereas *Nestin::Cre;Kif3a^fl/+^*, *Nestin::Cre;Ift88^fl/+^* and *Nestin::Cre;FoxG1^fl/+^* mice were used as controls. We also crossed *Kif3a^fl/fl^ Centrin-2^GFP/GFP^* mice with *Kif3a^ko/+^ Nestin-Cre^+/−^* mice to generate conditional mutants with GFP^+^ centrioles. The mice were maintained on a C57Bl6/J background. E0.5 was defined as noon on the day the vaginal plug was detected.

### Immunofluorescence, *en face* views and *in situ* hybridization

Mouse brains were fixed by immersion in 4% paraformaldehyde at 4°C for 4 h (embryonic brains) or overnight (postnatal brains) then washed several times in PBS. Coronal sections (80 or 100 µm) were cut on a Vibratome (S1000, Leica). For *en face* views, telencephalic hemispheres were first isolated in DMEM/F12 (Life Technologies) and fixed in 4% paraformaldehyde (PFA) in PBS for 1 h at 4°C. Tissues were then washed in PBS and immunostained as described below. Before mounting, the ventral telencephalon was removed with Pascheff–Wolff microscissors on a silicone rubber plate, and the dorsal telencephalon was flat-mounted by making a few cuts around the edge.

For immunostaining, samples were blocked for 30 min to 1 h in PBS containing 0.1% Triton X-100 and 10% fetal bovine serum, and then incubated overnight with the primary antibodies. The following antibodies were used: rabbit anti-activated caspase-3 (1:1000, Cell Signaling, 9662), rabbit anti-ZO-1 (1:100, Life Technologies, 402200), rat anti-ZO-1 (1:100, Developmental Studies Hybridoma Bank, AB2205518), rabbit anti-Ninein (gift from Michel Bornens; Mogensen et al., 2000), rabbit anti-Tbr2 (1:500, Abcam, ab23345), rabbit anti-Adenylate cyclase 3 (1:500, Santa Cruz, sc-588), mouse anti-Arl13b (1:500, NeuroMab, 75-287), mouse anti-N-cadherin (1:100, BD Biosciences, 610921), rabbit anti-phospho-Histone H3 (Ser10) (1:500, Millipore, 06-570), mouse anti-Vimentin (phospho S55) [4A4] (1:1000, Abcam, ab22651; Weissman et al., 2003), rabbit anti-Pax6 (1:1000, Millipore, AB5409), rabbit anti-Ctip2 [25B6] (1:1000, Millipore, ab18465), mouse anti-β-catenin (1:500, Millipore, 05-665), mouse anti-acetylated tubulin [6-11-B1] (1:500, Sigma-Aldrich, T6793), mouse anti-γ-Tubulin [GTU-88] (1:500, Sigma Aldrich, T6557), mouse anti-Actin (1:100, Millipore, mab1501), rabbit anti-phospho-S6 ribosomal protein (1:100, Cell Signaling, 2211), rat anti-CD24 (1:100, BD Pharmingen, 557436) and species-specific Alexa Fluor 488, 594 and 647 (1:1000, Life Technologies) secondary antibodies. Tissues were counterstained with DAPI (Sigma), mounted in Fluoromount (Southern Biotech) and examined with a confocal fluorescence microscope (SP5 Leica) or a fluorescence microscope (Zeiss Observer.Z1 with Apotome and Hamamatsu camera). For TUNEL staining, coronal cryostat or vibratome sections were labelled with In Situ Cell Death Detection Kit according to the manufacturer's instructions (Roche).

For quantification of cells expressing Ctip2, PH3, Tbr2 or Pax6, a single confocal fluorescent optical section was analyzed. A rectangle with a fixed width (220 µm) spanning the entire thickness of the cortex was placed at a 45° angle to the cortex to approximately mark the somatosensory cortex. Six regions per coronal section on a total of six sections at different rostro-caudal levels were analyzed for each condition. Quantification results are shown as indicated in graphs as a number per unit area (see Fig. 1E and Fig. 3G-J, Fig. S3E-F).

Anaphase spindle orientations were measured on reconstituted *Z*-projections from *en face* view acquisitions, using PH3 and γ-tubulin to identify anaphase and pericentriolar material, respectively. The anaphase angle was the angle between the vector connecting the two anaphase-centrosomes and the vector connecting apical surface centrosomes. For *in situ* hybridization, E12.5 mouse brains were fixed overnight in 4% PFA at 4°C. 100 µm vibratome sections were hybridized as described (López-Bendito et al., 2006) with digoxigenin-labeled *Ngn2* and *Dbx1* probes (Lu et al., 1994).

### Segmentation

Semi-automated segmentation of ZO-1 was performed with Packing Analyzer software (Aigouy et al., 2010), which allows manual correction after automated detection. Segmented images were then vectorized in Illustrator CS6 (Adobe). Finally, images were analyzed with CellProfiler software (Lamprecht et al., 2007) to generate data and color-coded area maps. Edges of the picture were excluded from the analysis.

### Scanning and transmission electron microscopy

For scanning electron microscopy, brain embryos were dissected in PBS and fixed in 2% PFA/2.5% glutaraldehyde. Fixed samples were treated with 2% osmium and washed several times in ultrapure water, dehydrated in a graded series of ethanol concentrations and prepared for scanning electron microscopy using the critical point procedure (CPD7501, Polaron). Their surfaces were coated with a 20 nm gold layer using a gold spattering device (Scancoat Six, Edwards). Samples were observed under a Cambridge S260 scanning electron microscope at 15 keV.

For transmission electron microscopy, dorsal E12.5 telencephalons were dissected in PBS then fixed in a 2% PFA/1% glutaraldehyde in 0.1 M phosphate buffer for 3 h. After rinsing in PBS, the tissue was postfixed in 1% osmium tetroxide for 30 min on ice, protected from light, with shaking. The tissue blocks were then dehydrated in 50% and 70% ethanol baths for 7 min each then stained in 1% uranyl acetate in methanol. After the final dehydration, the samples were immersed for 40 min in a graded series of ethanol/Epon solutions (2:1, 1:1, 1:2 ratios), then in pure Epon. The samples were mounted in Epon blocks for 48 h at 60°C to ensure polymerization. Ultrathin sections (70 nm) were cut sagittally on an ultra-microtome (Ultracut E; Leica) and analyzed with a transmission electron microscope (Technai 12, Philips).

### Western blotting

Dissected dorsal E12.5 or E14.5 telencephalons were frozen in liquid nitrogen; four or five of the same genotype were pooled. Tissue was homogenized in RIPA lysis buffer (50 mM Tris-HCl pH8, 150 mM NaCl, 1% Igepal CA-630, 0.5% sodium deoxycholate, 0.1% SDS) with Complete ULTRA protease inhibitor cocktail (Roche) and PhosSTOP phosphatase inhibitor cocktail (Roche). The homogenates were centrifuged at 13,500 ***g*** for 30 min at 4°C and the clear lysates stored at −80°C until used. Total protein content was quantified with the BCA protein assay (Thermo Fisher Scientific). Samples were then diluted in 2× Laemmli SDS Sample buffer (BioRad) and boiled for 5 min at 95°C. Equal amounts of protein (50 or 100 µg) were run on precast 7.5% Mini Protean TGX Gels (BioRad) and transferred overnight at 4°C onto nitrocellulose membranes (BioRad). The membranes were blocked in 5% non-fat dry milk in TBS-T (pH 7.4 with 0.1% Tween 20) for 1 h at room temperature. Primary antibodies were diluted in 3% BSA (Sigma Aldrich) and the membranes were incubated overnight at 4°C. We used the following antibodies (1:1000 dilution, from Cell Signaling unless otherwise indicated): rabbit anti-phospho-mTOR (Ser 2448) (2971), rabbit anti-mTOR (2972), rabbit anti-p70S6 kinase (9202), rabbit anti-phospho-p70S6 kinase (9205), rabbit anti-S6 ribosomal protein (2217), rabbit anti-phospho-S6 ribosomal protein (4857), mouse 6F5 anti-Gli3 (1:500, Genentech), mouse anti-GAPDH (1:10,000, Ambion). After removing the primary antibodies, the blots were washed several times in TBS-T then incubated with horseradish peroxidase-conjugated secondary antibodies (1:10,000, Jackson ImmunoResearch). Secondary antibodies were detected by SuperSignal West Dura or West Femto Maximum Chemiluminescent substrate (Thermo Scientific). Signals were analyzed with an ImageQuant LAS 400 device (GE Healthcare). Unsaturated bands were quantified using ImageJ (http://rsb.info.nih.gov/ij).

### Rapamycin treatment

Rapamycin (Merck) was dissolved in 100% ethanol, stored at −20°C and diluted in vehicle containing 5% Tween 80 and 5% PEG400 (Sigma-Aldrich) just before injection. For *in vivo* administration, pregnant E12.5 dams received an intraperitoneal injection of rapamycin (1 or 6 mg/kg) or vehicle.

### Statistical analysis

For normally distributed data (data shown as the mean±s.e.m.), a two-tailed Student's *t*-test was performed. For nonparametric distributions (data shown as the median), a two-tailed Mann–Whitney test was performed. *P<0.05, **P<0.01 and ***P<0.001.

Area of the lateral ventricle was measured by outlining the ventricle of three representative sections and the mean value plotted on the graph (*n*=3) (Figs 1 and 6).
